# Using Transparent Soils to Observe Soil Liquefaction and Fines Migration

**DOI:** 10.3390/jimaging8090253

**Published:** 2022-09-19

**Authors:** Jisun Chang, David Airey

**Affiliations:** 1 Arup, Sydney, NSW 2000, Australia; 2 School of Civil Engineering, University of Sydney, Camperdown, NSW 2006, Australia

**Keywords:** soil liquefaction, fines migration, mud pumping, cyclic liquefaction, wetting front, transparent soils

## Abstract

The cyclic liquefaction of soils and associated mud-pumping can lead to costly repairs of roads, railways, and other heavy-haul infrastructure. Over the last decade, several laboratory studies have been conducted to investigate these phenomena, but, due to the opacity of soil, the typical experimental observations of cyclic liquefaction have been limited to post-test observations of fine movement and the data of water pressures and soil settlements. In this paper, we show how partially transparent soil models can be used to provide the visualization of a moving saturation front and that fully transparent models can be used to observe fine migration during the cycling loading of a soil column. The changing saturation degree was tracked using a correlation between the degree of saturation, soil transparency, and grayscale image values, while particle movements of fines and larger particles were measured using a small number of fluorescent particles and particle tracking velocimetry. Another innovation of the work was in using mixtures of ethyl benzoate and ethanol as a low-viscosity pore fluid with the refractive index matching the fused silica soil particles. The benefits and challenges of these visualization tests are discussed.

## 1. Introduction

The response of sandy soils to cyclic loading has been very widely studied because of the significant effects on structures, slopes, embankments, and buried infrastructure caused by the liquefaction of the ground following earthquakes. These studies have included numerous element studies in triaxial and simple shear apparatus, shaking table tests and dynamic centrifuge tests as well as the development of highly sophisticated constitutive models. More recently, there has been interest in the response of partially saturated soils to cyclic loading because, during earthquake events, they have been associated with significant compression [1,2,3] and have been involved in liquefaction failures [4,5,6]. If a significant number of fines is present, the fines are known to decrease these materials’ drainage potential, resulting in cases where materials, containing gravel sized particles and that are used for reclaimed fill purposes, have liquefied during earthquakes [7,8,9]. The migration of fines from the subgrade into railway ballast and highway pavements due to train and car traffic cyclic loading conditions is also known to reduce the drainage potential of ballast and pavement material, resulting in ballast and pavement degradation and large settlements of the infrastructure above [10,11,12,13]. The flow of fines has also been widely investigated because of the importance of internal erosion in dams and dewatering and the grading, effective stress and hydraulic conditions for fines migration have been established [14,15,16]. However, despite the significance of the problems, attempts to visualize these phenomena are rarely reported. This is related to the challenges of visualizing sufficient material at a resolution and timing that will allow meaningful observations to be made.

Shake tables have been used to investigate the pore pressure responses and 1D settlements in sands [17,18,19,20]. In all of these shake table studies, increases in pore water pressures and significant settlements were measured once liquefaction had occurred. Some studies also measured and observed an upwards flow of water and the propagation of pore water pressures throughout the soil column towards the surface once liquefaction had occurred [19]. Some studies, including those performed by Kokusho and Kojima [21] and Ozener et al. [22], used shake table apparatuses to investigate pore water pressure generation in sand with silts. A recent study with significant fine contents by Kwa et al. [23] showed that partially saturated silty sand columns prepared at degrees of saturation ranging from 50 to 70% all liquefied if subjected to sufficient cycles and that complex behaviors involving settlement, pore pressure build-up, saturation front rise, and flow leading to fine migration to the surface were observed. In this paper, we discuss the use of transparent soil to observe and understand the phenomena reported in Kwa et al. [23]. We concentrated on observing the saturation front movement in initially partly saturated soil and the particle movements, including of fines <75 µm, during the shaking of saturated soil columns. To facilitate this work, we used novel low viscosity pore fluids with matched refractive indices to fused silica particles for transparency and fluorescent particles to observe individual particle motions.

To visualize the processes and to understand the mechanics occurring at different scales, studies have used a range of techniques (photography, γ-radiation, X-rays, magnetic resonance) to investigate multi-phase flow and particle migration [24,25]. Of the many techniques available, digital photography is the most suitable for imaging cyclic soil tests because of its relatively low cost and fast image rates. However, it has the disadvantage that model porous media are required, and experiments are generally limited to two-dimensional views, either because the experiment is intentionally 2D, because of averaging in the third dimension, or because limited transparency limits observations to those at the boundary. Werth et al. [24] noted that methods to measure transient processes, such as cyclic phenomena, and fluid and particle transport in porous media were in need of development.

Transparent soils, which make use of particles and pore fluids with matched refractive indices, have been used in a variety of geotechnical, flow, and contaminant migration studies [24,26,27]. They allow measurements to be made at high spatial and temporal resolutions for greater sample depths than are possible using light reflection or transmission techniques. They can also facilitate 3D measurements when used in conjunction with the laser illumination of multiple planes and with multiple cameras. Geotechnical-focused uses of transparent soils that use realistic soil such as particles and that study large numbers of particles in 3D include studies of multiphase flow [28,29] and of internal erosion and fines migration [30].

Sills et al. [29] described column experiments in which changing transparency was used to measure the degree of saturation as the head rises or falls. However, these experiments used fused silica and a mineral oil with a viscosity 10 times that of water. To correctly model transient flow that would occur during cyclic column loading, a fluid with a viscosity closer to water is required. Previous investigations have identified pore fluids with a low viscosity [26,31], but these are either expensive or present health risks, and alternative pore fluids that are both low cost, stable, and widely available are desirable.

Rosenbrand and Dijkstra [32] and Ouyang and Takahashi [33] used photographic methods to infer fine movements from changing patterns and colors at the boundary of soil containers, but, although the pattern of fine movement could be observed, no detail of the individual fine particles could be detected. As discussed by Rosenbrand and Dijkstra, both the movement of individual fine particles and the changing distribution of the fines are important, and this presents challenges with sample dimensions, resolution, and image acquisition rates, and the ability to track particles requires a relatively low concentration of fines, which prevents the study of the fine structure. Hunter and Bowman [30] used transparent soil to avoid boundary effects, with the visualization of soil planes made possible by a low concentration of fluorescent dye mixed into the pore fluid. However, owing to the viscosity of the pore fluid, soil particles had to be scaled up, and, although areas of fines could be observed, and despite the relatively large size of the fines, the motions of individual fine particles could not be reliably detected. Nevertheless, the tracking of individual fines has been performed in microfluid experiments by using fluorescing particles, with particles of clay size being observed [34], albeit for a very small field of view. The use of fluorescent particles in geotechnical scale problems has received little attention, although these have the potential to allow 3D movements to be directly measured [35].

To investigate both the saturation front movement in partially saturated soil and the particle movement during cyclic liquefaction events, experiments were performed using visualization techniques relying on soil transparency and fluorescing particles. This paper discusses the selection of low viscosity pore fluids, the experimental procedures, and the results of saturation front movement and fine movements for silt-sized particles of approximately 50 µm diameter. Suggestions for the future development of the methods are presented, which can support in turn the development of constitutive models that are being proposed to predict fine migration (e.g., Yin et al. [36]). These local measurements are important for validating numerical models of multiphase flow in porous media, as well as for linking observations to coupled processes, such as multiphase flow and the mass transfer between fluids.

## 2. Materials and Methods

This paper presents two methods utilizing transparent soil models to observe both the saturation front movement of an initially partially saturated column and the particle movements, including of fines <75 µm, during the shaking of an initially saturated soil column. The soil saturation profile was monitored using optical photography by adopting the method presented by Sills et al. [29], of calibrating 8-bit grayscale image values to soil saturation, whereas the migration of silt sized particles within the bulk of a fully saturated soil column was tracked by means of optical photography and the use of fluorescing particles.

### 2.1. Granular Material

**Figure 1:**
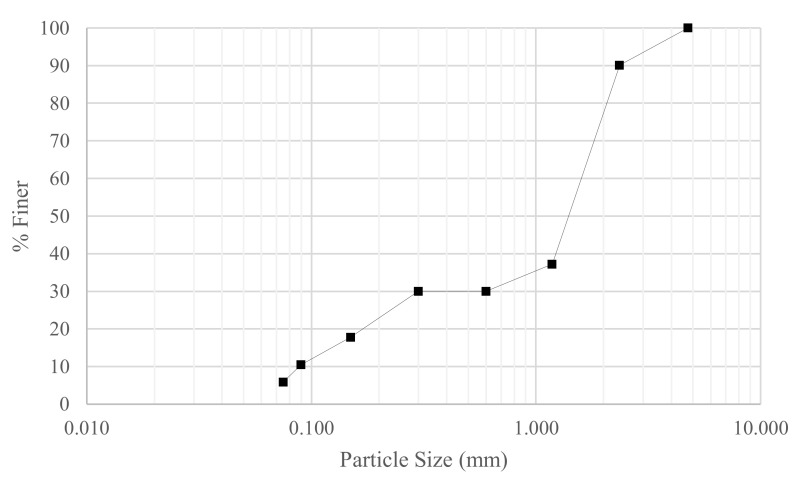
Particle size distribution.

The granular material was prepared from high purity fused silica (HPFS) with specific gravity, Gs = 2.2, and was comprised of a bimodal mixture of grain sizes, with 70% between 0.85–3 mm and 30% silica fines ranging from 150 μm to <75 μm. The grading curve is shown in Figure 1. Maximum and minimum void ratios were determined as 1.280 and 0.861, respectively.

Fluorescent-coated borosilicate glass beads 45–53 μm in diameter, Gs = 2.2, from Cospheric were used as fine tracer particles. Approximately 0.2 g of the particles was added to the soil model (mass fraction 0.01%; total volume fraction 0.005%, about 190,000 particles), which is a sufficiently high number of particles so that they could be observed in any sample of the soil whilst being few enough to be distinguished as individual particles. A small number (approx. 50, <1 g) of 425–500 μm diameter fluorescent-coated barium titanate glass beads from Cospheric were also included to trace the movement of large particles and, hence, the bulk mass of the soil.

### 2.2. Pore Fluid

The selection of a pore fluid with a matched refractive index (RI) to the soil grains (fused silica, *n* = 1.4586) is a key consideration when using transparent soil models. For the tests reported here, looking at both the saturation front movement and particle movements, the fluid density, kinematic viscosity, and surface tension should be close to that of water. The fluid also needs to be chemically stable and insensitive to changes in temperature and humidity. In addition, the fluid should be incompressible and affordable, present minimal hazard, and have no interaction with any materials or sensors with which it is in contact. Previously proposed fluids for geotechnical model tests with a viscosity similar to water include mixtures of toluene with methyl ethyl ketone and toluene with 2 propanol [31], but these present safety hazards and require special precautions. A more recently proposed alternative is to use sodium iodide solutions treated with sodium thiosulphate [37]; however, sodium iodide is expensive, and, to control costs, time consuming procedures are required to enable the solution to be re-used. We successfully used this fluid [38] and found that a 90% recovery is possible [39], but the time required for fluid handling processes with large geotechnical model tests and the reactivity of the iodide solution present considerable challenges, making this approach unsuitable for routine use. A wide variety of alternative pore fluids have been used for transparent soils, mainly in microfluid experiments, which were summarized by Wiederseiner et al. [40]. Based on a review of the literature, several potential pore fluids that do not present significant hazards were identified, and these are listed in Table 1. Preliminary tests identified issues in stability and maintaining transparency in some cases and led to the adoption of turpentine/ethanol and ethyl benzoate/lamp oil mixtures in the tests described below.

For the tests using unsaturated soil-fluid mixtures, ethyl benzoate and lamp oil were used for the pore fluid. Ethyl benzoate has not been used previously for transparent soils, though lamp oil (or mineral oil blends) is well established for use in conjunction with paraffin oil [26]. Ethyl benzoate and lamp oil were prepared at a ratio of 1:3 to achieve the required refractive index (*n*). The refractive index was measured using an ATAGO RX-5000 refractometer; then, either ethyl benzoate or lamp oil was added to correct the refractive index up or down, respectively. A batch of approximately 1.5 L was prepared to n = 1.45847. Pore fluid density was determined to be 0.866 g/cm^3^ at 25 °C.

Ethyl benzoate presents several advantages over the commonly used pore fluid mixture of paraffin oil and lamp oil in viscosity and reasonably low volatility. It has a high refractive index of 1.50328, a viscosity of 1.971 mPa.s at 21 °C [41], approximately twice that of water, a low vapor pressure, and a high flashpoint of 93.4 °C [42]. Lamp oil similarly has a low vapor pressure and high flash point (118 °C), which contribute to the evaporative stability of the pore fluid mixture [43]. Ethyl benzoate is also relatively non-toxic; it is commonly used as a component of artificial fruit flavors. The cost is modest at approximately AUD 150 per liter.

**Table 1:** Pore fluids considered for testing. Values are given for 21–25 °C.

Pore Fluid	Dynamic Viscosity (mPa.s)	Fluid Density (g/cm^3^)	Refractive Index	Hazard/Toxicity	Source	Initial Test Results
Water	0.89	0.997	1.333	N/A	N/A	N/A
Light Mineral Oil (e.g., Penreco Drakeol #5)	11.9	0.8449	1.4664	Non-hazardous.	Stoots et al. [44]	Not tested due to high viscosity.
Para-cymene + Ethanol	0.84 (Para-cymene) 1.18(Ethanol)	0.855 (Para-cymene) 0.789 (Ethanol)	1.488 (Para-cymene) 1.3614 (Ethanol)	Irritant in case of skin or eye contact. Avoid ingestion or inhalation.	Fort et al. [45]	Not selected in lieu of a less toxic pore fluid.
Cyclo-octane	2.996	0.834	1.4578	Avoid ingestion or inhalation.	Stephenson and Stewart [46]	Minor difference in RI with HPFS reduced transparency. Not selected in lieu of a less viscous fluid.
2,2′-thiodiethanol (TDE) + Glycerol + Phosphate Buffered Saline (PBS)	65.2	1.175–1.195	1.5217	Severe irritation if splashed into the eyes.	Zhu et al. [47]	Not tested due to high viscosity
Turpentine + Ethanol	1.49 (Turpentine) 1.18(Ethanol)	0.847 (Turpentine and Ethanol refractive index matched to fused silica)	1.47 (Turpentine) 1.3614 (Ethanol)	Irritant in case of skin or eye contact. Avoid ingestion or inhalation.	Cui and Adrian [48]	Used for fully saturated experiments.
Deep Eutectic Solvent Choline Chloride:Urea (aq), 10–20% concentration	1.460–2.481	1.197 (100% ChCL:U)	1.5056 (100% ChCL:U)	Inconclusive. The components urea and ChCl are non-toxic; some studies suggest synergistic increase in toxicity (Hayyan et al. 2013).	RI and viscosity data from Shekaari et al. [49]. Fluid has not previously been used in transparent soil models.	Solvent showed signs of crystallization one day after preparation. Fluid preparation took multiple days.
Sodium Iodide + Sodium Thiosulfate + Water	2.2	2.05	1.333–1.501	Non-hazardous.	Carvalho et al. [37]	Not tested due to time-intensive preparation and recovery process.
Sucrose + Water	201.2	1.985	Up to 1.503	Non-hazardous.	Guzman and Iskander [50]	Not tested due to high viscosity
Methyl Benzoate + Ethanol	1.851–1.918 (MB)	1.085 (MB)	1.514 (MB)	Edible. Irritant in the case of excess exposure.	Zisselmar and Molerus (1979) in Wiederseiner et al. [40] RI and viscosity data from Sheu and Tu [41]	Not tested in lieu of lower viscosity ethyl benzoate.
Ethyl Benzoate + Lamp Oil	1.971 (EB) 2.13 (C14 alk.) 2.86 (C15 alk.) 3.47 (C16 alk.)	1.013 (EB) 0.866 (RI matched to *n* = 1.4586) 0.7628 (C14 alk.) 0.7685 (C15 alk.) 0.7701 (C16 alk.)	1.5032 (EB) 1.4290 (C14 alk.) 1.4315 (C15 alk.) 1.4329 (C16 alk.)	Edible/skin respiratory irritant.	This paper RI and viscosity data from Sheu and Tu [41]	Not used for the fine migration experiments, as the mixture would degrade the polymethyl methacrylate (Perspex) column.

A high vapor density is important in maintaining a constant refractive index; otherwise, high rates of evaporation would cause the ratio of component pore fluids, and thus the overall refractive index of the pore fluid, to change over time. The experiments required the unsaturated soil, which had a large air-fluid interfacial area, to be manipulated for up to 1 h, and, thus, a very low evaporation rate was critical to ensure that the calibration of the image against an 8-bit pixel intensity scale remained stable.

The tests performed to observe fine migration were performed using fully saturated soil–fluid mixtures. For these tests, a 1:1 mixture of pure gum turpentine and ethanol was used for the pore fluid. Pure Gum turpentine was used together with 99.5% purity ethanol. The initial refractive index was measured using an ATAGO RX-5000 refractometer; then, either turpentine or ethanol was added to correct the refractive index up or down, respectively. Pore fluid density was determined to be 0.847 g/cm^3^ at 25 °C. The viscosity of the turpentine and ethanol mixture was 1.49 mPa.s, comparable to water (0.89 mPa.s).

Due to the high vapor pressures of turpentine and ethanol, well above atmospheric pressure, the mixture evaporated rapidly in an open container, and the slight difference in evaporation rates of ethanol and turpentine caused the particle-fluid mixture to lose its transparency. Preliminary tests of a fully submerged column indicated that the variation of the refractive index remained within the tolerable range of ±0.002 [51] for at least 20 min when a 1–2 cm thick layer of the pore fluid was maintained above the soil.

Despite the challenges of maintaining a precise refractive index, the ethanol and turpentine pore fluid mixture presents an attractive alternative for fully saturated applications due to its ubiquity and low cost.

One possibility to reduce the volatility of the pore fluid is to mix ethanol with water, as ethanol can dissolve approximately 10–20% *v/v* of water. This ternary mixture was not used in this study, but it remains a viable route for further investigation.

#### Chemical Compatibility

Ethyl benzoate is a strong solvent, capable of dissolving the majority of common plastics and polymers such as silicon sealants, PMMA, polycarbonate, and polystyrene [52], but it does not affect the HPFS grains. To prevent chemical degradation, a tall square glass jar 10 cm × 10 cm × 30 cm made from a single piece of glass was used for the sample column for the unsaturated column tests. All mixing and tamping equipment were made of stainless steel.

The turpentine and ethanol mixture may also dissolve or cause crazing to certain common types of plastics, particularly at elevated temperatures [52]. For the saturated column tests, the container was made of PMMA. The polymer material of the fluorescent particles was unknown, but no adverse effects were identified despite them being submerged in the pore fluid for more than 2 weeks. All other implements used were made of stainless steel.

### 2.3. Apparatus and Procedure

The experiments consisted of placing granular material—pore fluid mixtures—into transparent columns, which were secured to a small shaking table, and then activating the shaking table for a few minutes. The vibration caused fluid pressures to rise, the granular material to compress, and, when initially unsaturated, the saturation front to rise. Photography was used to record particle movements and to record changes in transparency. The shake table used in this study was designed to perform maximum density tests according to Australian Standard AS1289.5.5.1; it has a fixed frequency of 50 Hz and produces a regular pulsed waveform with peak vertical accelerations of approximately 0.4 g.

Slightly different procedures and apparatus were needed in the two experiments because of the different pore fluids and because of the quality of the photographs required, as explained next.

### 2.4. Experiment 1: Wetting Front Mobility 

A low volatility transparent soil model of high purity fused silica (HPFS) and ethyl benzoate/lamp oil pore fluid prepared to a nominally uniform saturation was placed on a shaking table against a dark background.

The unsaturated soil sample was placed into the column in 5 layers of equal volume and mass. Each layer was approximately 5 cm thick and was tamped down to a pre-determined void ratio, which in most tests was 1.1, a relative density of 43%, using a stainless-steel tamping block. Some settlement occurred during preparation, and the final average void ratios achieved, given in Table 2, were slightly lower than the target values.

**Figure 2:**
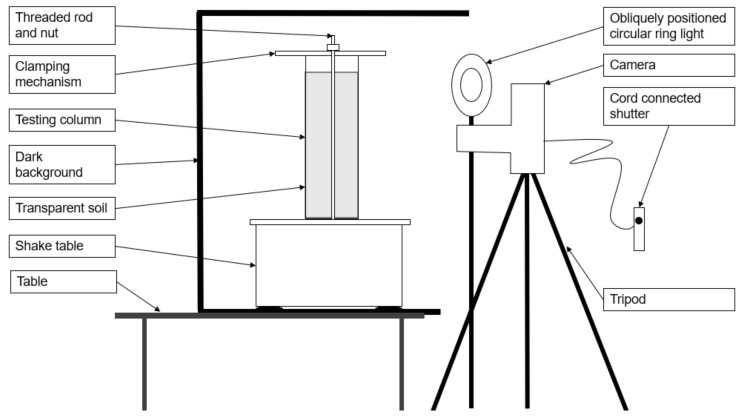
Experiment 1 setup—side elevation.

The glass column was clamped to the shaking table using a rubber padded timber top plate. The shake table and column were placed within a 5-sided black timber box, which provided a black backdrop and limited the light exposure to the back and sides of the soil column. A Canon EOS50 DSLR camera was mounted at mid-height of the column and isolated from the shaking apparatus to minimize vibrations from the shaking table. Care was taken not to disturb the placement of lighting and cameras as the column was removed and replaced between trials, as this would have necessitated a re-calibration of pixel intensity. A schematic of the experimental setup is shown in Figure 2.

The samples were shaken for a period between 1.5 min and 2.5 min. Images were captured either every 5 s or every second. Tests were run with initial saturations of 0%, 33%, 67%, and 100%. The series of experiments is tabulated in Table 2. The tests with 100% and 0% saturation were required to calibrate the pixel intensity to saturation relation [29].

#### 2.4.1. Photography

The method of analysis for this experiment is dependent on the pixel intensity of the images captured, and, consequently, a stable image brightness and contrast level were critical. The camera was configured to manual exposure, manual focus, a fixed lens aperture of 22, and a constant frame rate of 50.

A circular ring light was used to better illuminate the images and to reduce the reflections off background objects in the laboratory. Preliminary trials showed the reflection of the experimenter casting a shadow and hence altering the pixel intensity of the test. The ring light was positioned obliquely to avoid direct reflection of the light off the glass of the soil column.

#### 2.4.2. Digital Image Analysis

Images were imported to the Fiji image analysis software [53] as an image stack and converted to 8-bit grayscale.

The ROI Manager package in Fiji [53] was used to create equally-sized ‘cells’ 50 pixels high along the height of the column. The cell width was adjusted so that only the central width of the column was captured, taking care not to include the curved edges of the jar where reflection and brightness were inconsistent. The software add-in Multi Measure [54] was used to measure the average pixel intensity of each cell across the entire time series.

Upon initial analysis of the uniformly saturated Test 2, a ‘bulge’ in pixel intensity was found along the height of the column due to the location and shape of the ring light. A subtraction array was determined during post-processing to correct for the uneven illumination. Average pixel intensities were normalized then converted to saturation using the relationships proposed by Sills et al. [29]. Average pixel intensities were normalized as follows:

(1) $$I_{N}=\frac{I_{D}-I}{I_{D}-I_{S}}\;$$

where

*I_N_* is the normalized pixel intensity between 0 (dry) and 1 (saturated);

*I_D_* is the average pixel intensity for a completely dry column;

*I_S_* is the average pixel intensity at 100% saturation;

*I* is the pixel intensity of the segment being measured.

A simplified relationship between fluid saturation and pixel intensity was then used to determine the fluid saturation in each cell.

(2) $$S_{w}=\left(\frac{S_{r}-1}{\ln\left(I_{Nr}\right)}\right)\ln\left(I_{N}\right)+1$$

where

*S_r_* and *I_Nr_* are the saturation and average pixel intensity at 33% saturation;

*S_W_* and *I_N_* are the saturation and average pixel intensity of the cell under analysis.

Whilst the sample did become markedly darker when saturated, it did not become completely transparent. The normalization of pixel intensities, which remove dependence on absolute pixel intensity values, was assumed to correct for this error.

### 2.5. Experiment 2: Particle Tracking in Saturated Soil Columns

Particles that fluoresce under UV light were used in a saturated transparent soil mix of HPFS grains and turpentine-ethanol pore fluid to trace the movement of both fine and large particles. Images were captured using macro photography; then, a particle tracking velocimetry software, TrackMate [55], was used to determine the velocity vectors of the particles across each frame.

#### 2.5.1. Experimental Details

To prepare the soil columns, the HPFS was mixed with the pore fluid, ensuring that the particles were always submerged. Due to the low viscosity of the ethanol-turpentine mixture, bubbles were easily removed by agitating the sample container. The saturated mixture was then placed in the column in 5 equal layers, approximately 5.5 cm thick, and tamped to produce a void ratio of e = 1.0 (relative density of 67%). A layer of pore fluid of 0.5–1.0 cm was maintained above the top of the particles. A lid was placed on the column after filling to reduce the amount of evaporation. The test column base was then bolted to the shake table.

**Figure 3:**
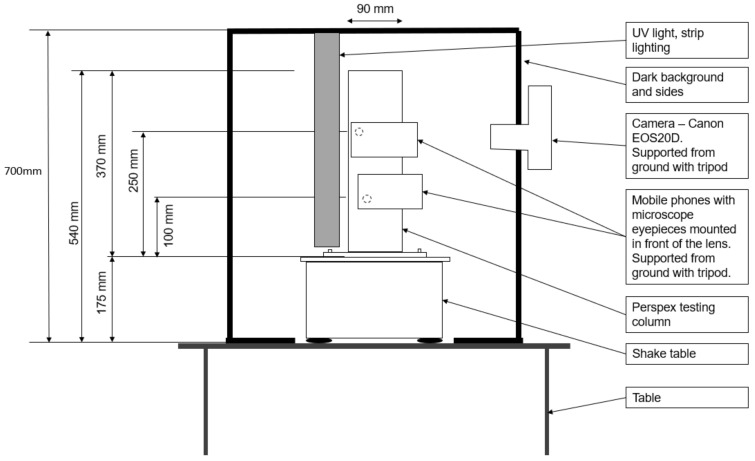
Experiment 2 setup—front elevation.

**Figure 4:**
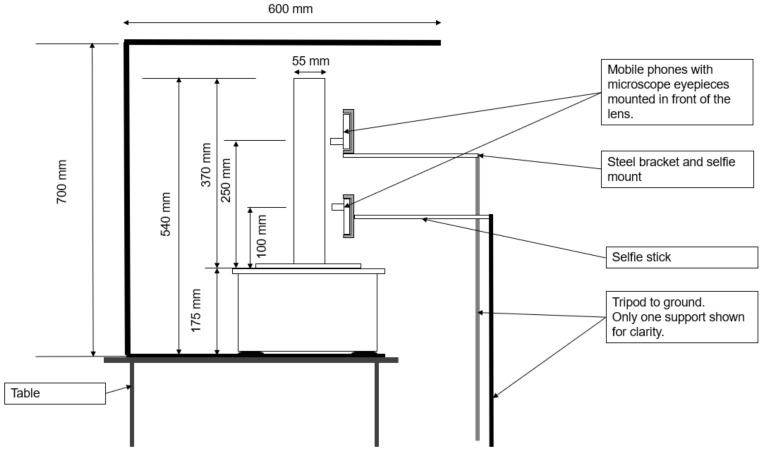
Experiment 2 setup—side elevation.

A 40 cm long UV strip-lighting bar was vertically suspended parallel to the column; its position was adjusted to maximize the glow of the fluorescent particles. The shake table and UV lighting were placed inside a black-painted timber box to minimize interference with background light sources. The front face of this box was left open to enable camera access, as shown in Figure 3 and Figure 4.

The test procedure involved activating the shaking table for 1 s in every 5 s for an approximate duration of 1000 s. The pulsing of the shake table was necessary to enable clear images of the fine fluorescent particles to be obtained during periods when the table was stationary. When the shaking was applied for longer than 5 s, the displacements of many particles were beyond the capacity of the software to track; the search radius in the tracking software would need to span the height of the image, and the identical appearance of the fluorescing particles would yield traces between two different particles.

#### 2.5.2. Photography

Three cameras were used to photograph the experiment. Two macro images approximately 10 mm in diameter were taken at elevations of 10 cm and 25 cm. Two smartphones with microscope eyepieces at x10 magnification were mounted in front of the phone cameras to view the 45–53 μm particles. The open-source mobile camera application OpenCamera [56] was used to take an image every 5 s. The lenses were placed approximately 5–10 mm away from the Perspex column surface. Each view was framed on one side with tape annotated with graduations to determine the pixel-to-mm scale during post-processing.

A Canon EOS50 DSLR camera was used to capture the movement of the larger particles, visible from the sides of the column where its view was not obscured by the mobile phones and was programmed to capture an image every 5 s.

Settings for all three cameras were set to manual ISO exposure, shutter speeds, and focal length. Fixed exposure was required to ensure consistent color thresholding during post-processing. A fixed focal length was particularly important for the macro images, as the short focal length meant that only particles within a narrow slice (<2 mm) were brought into focus. Shutter speeds were set to 50 (i.e., 0.02 s exposure length) for consistency in the brightness of the images. All images were taken in color to ensure that the green, fluorescent particles could be isolated during post-processing.

#### 2.5.3. Post Processing

Photos from each camera were imported as image stacks. Images were scaled down to 25% or less due to the large file sizes, translating to 200–400 MB per stack. Color thresholding [57] was used to distinguish the green, fluorescent particles, and the threshold images were converted to 8-bit greyscale.

The TrackMate plugin [55] was used to determine the path and average velocity of the particles. The search radius for potential tracks was set to between 5–7 times the average particle diameter (typically 10 pixels), and any tracks with displacements larger than this were hence excluded, as were tracks that could not be followed across 3 consecutive pictures. For macro images, links in tracks were only made between consecutive frames. Non-consecutive frame searches were used when tracking the larger particles, as relative displacements were small, and the individual particles could be clearly identified. The applications of the above filters and parameters eliminated several high velocity links but were required to reduce the number of wild tracks.

## 3. Results

### 3.1. Experiment 1—Wetting Front Mobility

**Figure 5:**
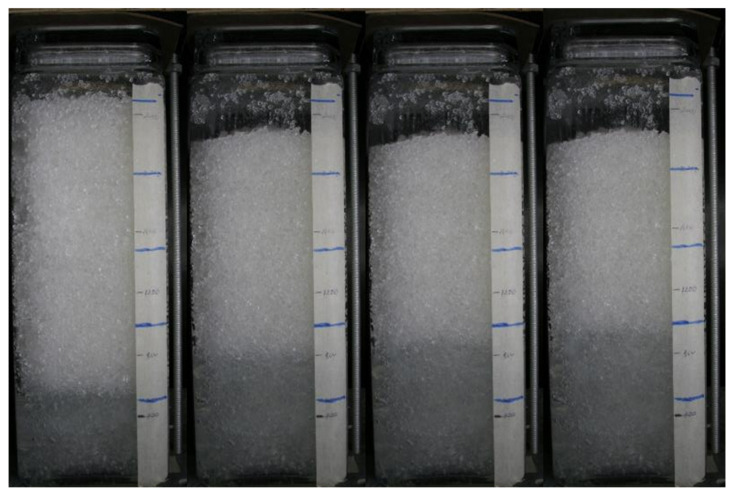
Sample image sequence from test 5 taken at time intervals t = 0 s, t = 30 s, t = 60 s, and t= 90 s.

**Figure 6:**
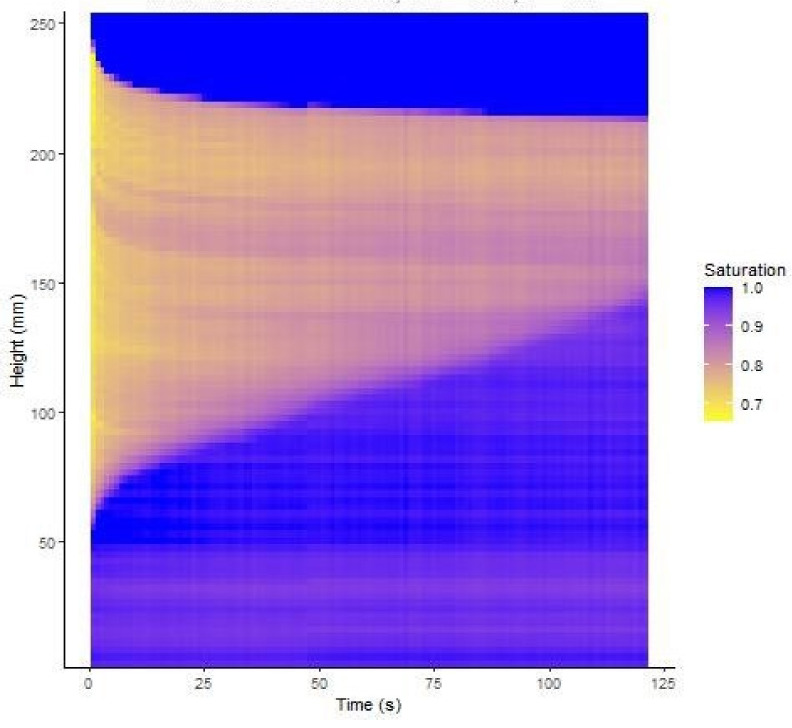
Evolution of saturation degree with depth in test 8.

A typical set of photographs from test 5 are shown in Figure 5. It can be seen that, before starting to shake the column, a darker, almost saturated region developed at the base of the column, indicating that some pore fluid redistribution occurred following the initial compaction procedure. After 30 s, significant settlement of the upper surface was evident as well as a rise of the saturation front at the base of the column. Further shaking led to little additional settlement, but the saturation front continued to rise. Above the upper surface of the soil, the black background was revealed as the soil settled which led to the algorithm indicating an apparent saturation of 100%. It may also be noticed that the exact level of the upper surface of the soil column is difficult to determine precisely because some HPFS particles stuck to the sides of the container, and the settlement was not uniform across the surface. This uncertainty affected the accuracy of the average degree of saturation inferred from the measured masses of solids and fluid, which were closely controlled. The degree of saturation estimated from the analysis of the pixel intensities for test 8 is shown in Figure 6. This figure puts together results from all the photographs taken at 1 s intervals throughout the test. This figure shows the initial rapid drop of the soil surface and the gradual rise of the saturation front throughout shaking with relatively little noise in the data.

**Figure 7:**
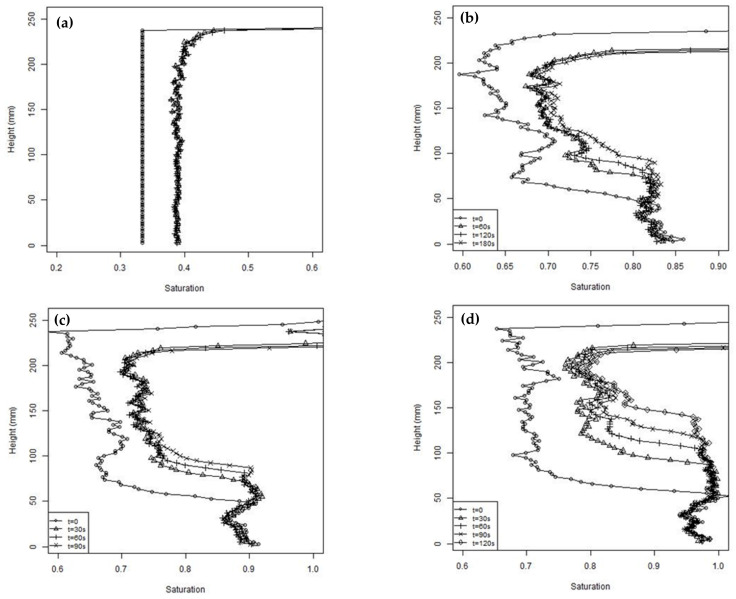
Saturation profiles at different times during the unsaturated tests (**a**) test 2, (**b**) test 3, (**c**) test 5, and (**d**) test 8.

Figure 7 presents profiles of the degree of saturation (S), with the depth at selected times after the commencement of shaking for four tests. Test 2, shown in Figure 7a, had an initial average degree of saturation of 0.33, whereas all the other tests had the same average initial S of 0.68. The relatively dry soil in test 2 was prepared to an average S = 0.33. The initial distribution was assumed to be uniform and was used to provide the subtraction array to correct all the subsequent photographs. Within a few seconds of starting the cyclic loading, the soil settled and reached S = 0.38, and after this, no further settlement or change in S was detected. Some apparent variation in S close to the top surface is indicated in Figure 7, which is believed to be a consequence of the limitation of using the initial subtraction array when the column has settled in addition to the issues mentioned above relating to the position of the top surface. The uniform increase in S suggests a uniform strain occurred throughout the column. If it is also assumed that no moisture migration occurs, and the settlement is one dimensional then the height change will be given by

(3) Δh=ei hi 1−SiSf1+ei

where *e_i_* = initial void ratio, *h_i_* = initial column height, *S_i_* = initial saturation, and *S_f_* = final saturation.

Using the estimated S values, Equation (3) predicted a settlement of 14 mm in test 2, whereas the measured settlement was approximately 7 mm. A settlement of 7 mm would be produced by a change in S of 0.021. It is believed this discrepancy was a consequence of changes in ambient light, the blurring of the photographs due to the cyclic loading affecting the measured pixel intensity, and the difficulty of accurately measuring the surface settlement.

Apart from test 2, all the other tests shown in Figure 7 were prepared with a nominally uniform degree of saturation of 0.68. Some variability of S with depth was expected because of the compaction method used in preparing the columns. However, it is clear from Figure 7 that, before starting cyclic loading, rapid pore fluid migration occurred during the few minutes taken to set up the apparatus, with pore fluid flowing to the base. A limiting S, of approximately 0.85, was observed over the lower 50 mm of the column in all the tests. The limiting saturation was less than 100% due to air bubbles being trapped by the soil fines. The pattern of the degree of saturation with depth was consistent with that expected of gap graded soils, such as those used in this study, which have bimodal soil water retention curves [58]. It can also be observed that the initial S values increased slightly as the tests proceeded. These tests were performed over one day, with the soil being reconstituted between each test. It is believed the change in initial S was an artefact caused by the change in the ambient lighting, which affected the pixel intensity, and possibly by a gradual loss of fines.

**Figure 8:**
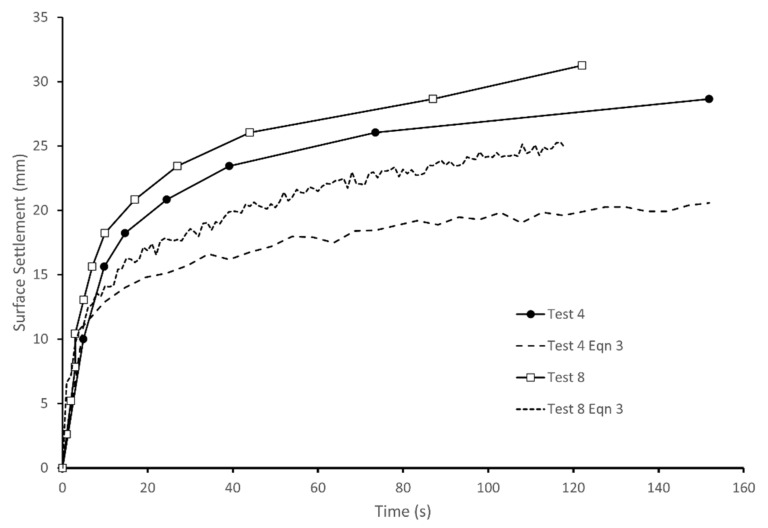
Surface settlements measured in tests 4 and 8 and estimated from changes in S.

During cyclic loading, all the tests show a similar pattern of settlement and an increasing degree of saturation in the upper part of the column and a rising saturation front at the base. The surface settlements estimated from the pixel intensity measurements are shown in Figure 8 for two typical tests. Figure 8 also includes estimates of the surface settlement calculated using a form of Equation (3) to sum up the effects of changes in S throughout the column when divided into 96 sub-layers. Figure 8 shows that the settlements estimated from Equation (3) agreed well with the measured values in the first 5 s. This indicates that the initial settlement was due to expulsion of air from the upper partially saturated region of the column, with the resulting reduction of void space leading to the increase in S, similar to the process occurring in Test 2. However, unlike test 2, the settlements continued throughout the test and diverged from those estimated from Equation (3). These additional settlements represent about one third of the total settlement, which was 26.6 mm on average from the five tests, with a nominal initial S of 0.68. The remainder of the settlement was due to upward migration of the pore fluid and associated reductions in voids ratio. As a result of the increase in S in the upper part of the column, the soil will not be in equilibrium and pore fluid migration will occur, leading to rise of the saturation front. At the same time, the excess pore pressures will rise in the saturated base due to the cyclic loading and cause an upwards flow of pore fluid, also contributing to the rise of the saturation front. The lag in settlements related to fluid flow can be explained by the time required for fluid pressures to build up, and the relatively slow rate of fluid drainage. After 30 s, the rate of settlement reduced and subsequent settlements were believed to be related to the dissipation of fluid pressures, which occurred at a slow rate through the partially saturated column.

### 3.2. Experiment 2—Fines Migration

After the preparation of the column, a layer of fine particles approximately 25 mm thick was observed at the top of the column, beneath a 20 mm layer of pore fluid. The fine layer was apparent from a slight reduction in transparency, and this was confirmed post-test by the absence of larger grains. The soil layer was 270 mm thick. It is believed that the fine layer was the result of excess pore pressures generated during compaction, causing an upwards flow of fines. At the end of the experiment, the fluid layer was 49 mm thick, the fine layer was 45 mm thick, and the soil layer was 223 mm thick. A repeat experiment gave layer thickness values within ±2 mm. These results indicate that the cyclic loading led to the densification of the soil layer, and this was accompanied by a loss of fines, which migrated upwards. By making reasonable assumptions for the densities, it can be estimated that the fine content decreased from about 27% to 23% in the soil layer. Similar movements of fines towards the surface were reported in other studies of columns subjected to cyclic loading [12,23].

**Figure 9:**
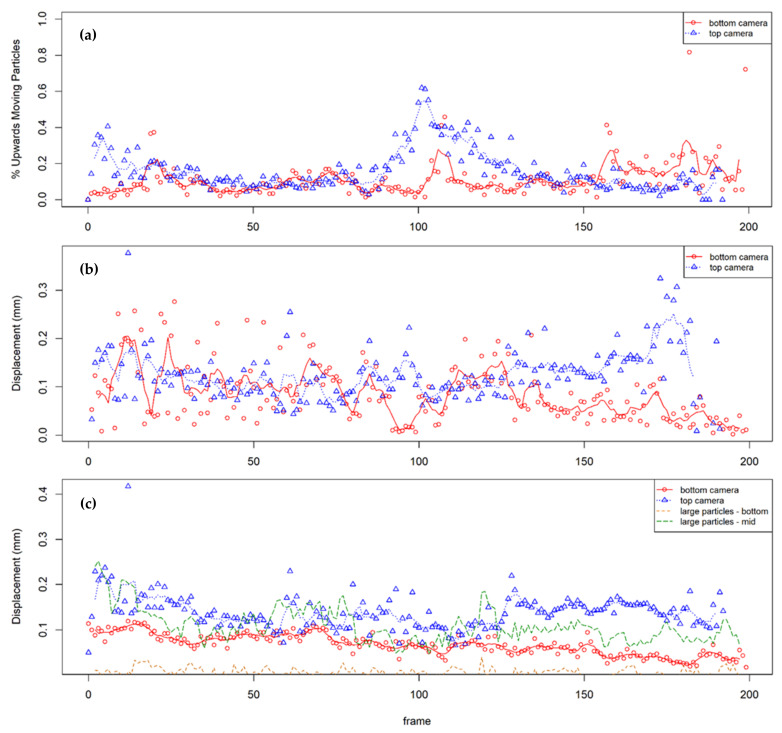
Analysis of average particle movement between successive photos: (**a**) % of upwards moving particles, (**b**) average upwards displacements, and (**c**) average downward displacements.

By observing the paths of the fluorescent particles, it was found that the large particles move predominantly vertically downwards as the soil layer compresses with greater movements near the top of the column, as would be expected. The fine particles were less constrained in their movements. To characterize their behavior, graphs are shown (Figure 9) indicating the percentage of upwards moving particles, and the displacements of the upwards- and downwards-moving particles between successive photographs, plotted against frame number (the photographs were taken at 5 s intervals). These measurements are presented for the two micro cameras observing fines at column elevations h = 100 mm (bottom camera) and h = 250 mm (top camera). Owing to the limited field of view and the similar appearance of the fluorescing particles, particles could not be tracked from one camera to the next. The downward movements of selected large fluorescent particles observed by the macro camera, which imaged the whole column, are included in Figure 9c for comparison. Any particles with no displacement, of which there were very few, were considered conservatively as downwards moving. Trendlines indicate the moving average. Whilst each point was captured after 1 s of vibration, the images were taken every 5 s.

The figures show that most of the particles were moving down with the compressing soil mass; however, significant amounts of the fines were moving upwards, and some of the upwards displacements were significant. The overall results are consistent with the increasing fine content at the surface, and these are discussed in more detail below.

In the first 20 pictures (t < 100 s), up to 40% of fines were recorded as moving upwards at the top camera (h = 250 mm). This may have been due to the initial consolidation of the upper layer and the subsequent displacement of pore fluid and fine particles. It may also be due to initial turbulence, which is supported by the highly variable rates of displacement in the same time period. The oscillating upward displacement at the base of the column was more pronounced than at the top; the regularity may suggest that this is mimicking the oscillation of the shake table. However, this movement was not mimicked by the downwards displacements, suggesting that fines may be partially in suspension and are being ‘pushed up’ with the pore fluid as it is displaced.

Between pictures 50 and 80, the percentage of upwards moving particles remained steady at 10–20%, suggesting a steady downwards consolidation of soil (i.e., 80–90% are steady or moving downwards). This is consistent with the increased rate of downwards displacement of fines at the top of the column.

Between pictures 80 and 140, there was a pronounced peak in fines moving upwards, first at the top of the column, then about 10 photos later, at the base of the column. This coincides with a moderate peak in the rate of upwards displacement. It appears the cameras captured a sand boil or a section of a homogenous upwards movement of fines. The movement of fines at the top first, then the bottom, echo the observations of Brennan [59], who noted in a 2D plane of view that the movement of silt particles was observed closer to the surface first, then in lower layers.

From picture 140 onwards, the velocities of the fine particles at h = 250 mm increased. As the tests progressed, the upper part of the column was increasingly made up only of very loose fine material, and the top camera was centered on this layer (see Figure 4). The resistance to movement in these layers was expected to be low, enabling consistently high velocities.

## 4. Discussion

The use of soil transparency as a measure of degree of saturation provided detailed information about the processes occurring during cyclic loading that would not be otherwise obtainable. The degree of saturation is normally estimated from measurements of gravimetric moisture content and density of small sub-samples or from buried moisture and displacement sensors. These measurements provide averaged values over relatively large volumes compared to the soil transparency technique. The repeatability and broad agreement of the measured saturation values above the wetting front with the calculated average values for the column provide confidence that the experimental method is valid. Although reasonably repeatable results were obtained, it is believed that, with improved calibration and control, the technique is capable of even more accurate results.

A single subtraction array was applied to all the photographs to account for the decrease in pixel intensity at the sample ends when the saturation is constant. Additional calibration checks to determine how this is affected by sample height and changing pixel values would improve the reliability of the inferred degree of saturation, particularly near the ends. An alternative lighting arrangement that more uniformly illuminates the column will also reduce the need for a correction in pixel intensity. The ethyl benzoate and lamp oil mixture maintained its refractive index throughout the series of experiments and performed well. However, the mixture has a viscosity just over double that of water, and for model experiments with unsaturated soil where flow is important, attention to scaling laws will be required. Another aspect that influences unsaturated soil behavior is the surface tension and wetting angle. As the surface tension of the organic fluids is less than for water, capillary rise will be reduced, and results will be representative of a more coarse-grained soil-water mixture. Further investigation to find other fluids that have a refractive index identical to fused silica and are both low-cost, non-toxic, and have a density, viscosity, and surface tension close to water is recommended. Suitable fluids are discussed in the review of Wright et al. [60].

One of the objectives of the experiment was to improve the understanding of similar unsaturated column tests reported by Kwa et al. [23], which used normal opaque soil and had moisture sensors, pressure transducers, and displacements measured at the column periphery. Although very different soil gradings were used in the two studies, the general pattern of the response of rapid settlement and the slow rise of a saturation front were observed in both. The full height coverage and near continuous record from photography using the varying transparency provided a more complete picture of the processes occurring than the isolated measurements taken at points on the boundary in conventional column tests [12,23]. The photographic evidence confirmed the settlement and moisture distribution mechanism suggested in Kwa et al. [23].

If there is no fluid migration, changes in degree of saturation can be used to calculate displacements, which was shown to provide reasonable estimates in the early stages of cyclic loading. However, when flow occurs, some means of measuring local hydraulic gradients and measuring fines transport are required to fully quantify the response.

The second experiment demonstrated that the tracks of fine particles could be followed; however, the method required a fully transparent soil column to allow the silt-sized fluorescent particles to be observed. In addition, high magnification was required, limiting the field of view and making it impossible to track particles travelling large distances in the 5 s between photographs. Some particles were observed travelling upwards 5–10 mm between each 5 s interval. Due to their speed, they were either not in the frame when the image was taken or were captured as blurred streaks in one frame only, but at least two frames were needed for tracking to occur. These large upwards movements were localized even within the small field of view and did not persist. The pattern suggested the development of temporary pipes of preferential flow.

The fluorescence of the fine particles was required for their identification, but, because the fluorescent fine particles were all similar, it was sometimes difficult to track them between photographs. Particles were also hemi-spherically coated, and due to the rotation of particles, the captured shape ranged from circles to semicircles; sometimes they were not captured at all if the coated face of the particle was turned away from the UV light source. As the tracking software used radius and particle shape in part in determining correlation, some links may have been incorrectly made or not made at all. The difficulty of identifying the particles could be improved by adding fines with a different color fluorescent coating.

One of the main limitations of the fine particle detection was the small field of view captured in this study. For a more detailed and quantitative study of particle motion, one or multiple cameras with a greater field of view are needed. Nevertheless, despite the limitations in this study, the ability to capture fine migration in conventional laboratory scale geotechnical experiments 2qw demonstrated. This technique has great potential to provide new insights into a range of internal erosion problems in geotechnical engineering.

## 5. Conclusions

Soil columns with varying initial degrees of saturation were subjected to cyclic loading and photographic methods used to observe settlements, saturation degree, and particle movements. The columns were comprised of high purity fused silica and pore fluids with matching refractive indices to provide transparent materials when fully saturated.

Tests with initially unsaturated materials using a pore fluid of ethyl benzoate and lamp oil were used to study how the degree of saturation in the column varied as a result of cyclic loading. The results showed an initial rapid settlement and increase in degree of saturation in the unsaturated soil followed by a gradual rise of a saturation front from the base of the column. The use of varying transparency as an indicator of degree of saturation was demonstrated to work effectively and has great potential for the experimental study of saturation changes in physical geotechnical models.

Tests of saturated transparent columns using a pore fluid of ethanol and turpentine were used to study particle movements during cyclic loading by placing a small number of fluorescent particles of 50 μm diameter in the columns. The movements of fine particles were successfully detected and showed that, while the general movement of the particles was downwards in the settling soil column, a significant number of particles could be observed moving upwards, and this was consistent with the accumulation of fines at the column surface. The ability to observe the movements of fines in geotechnical model scale tests as demonstrated here will have application in a range of geotechnical problems, most significantly those investigating internal erosion.

## Figures and Tables

**Table 2:** Tests conducted for wetting front mobility using the ethyl benzoate/lamp oil mixture.

Test	Saturation	Void Ratio	Analysed
1	30%	1.0	No
2	33.5%	0.975	Yes
3	68.3%	0.966	Yes
4	67.6%	0.975	Yes
5	67.6%	0.975	Yes
6	100%	1.1	Yes
7	67.6%	0.975	Yes
8	67.6%	0.975	Yes
9	0%	0.975	Yes

## Data Availability

Please contact the authors for experimental data.
